# The Training of Probationers

**Published:** 1917-03-17

**Authors:** 


### THE MATRONS' AND SISTERS' DEPARTMENT.

### THE TRAINING OF PROBATIONERS.

### The Process of Reconstruction and Hospital Education.

The theoretical training of probationers in all the various branches of hospital work is giving cause for much anxious thought to those who desire a higher standard of general efficiency in these days when so many professions are opening their doors to women of education. Unless hospital training can offer like advantages to the mental capacity of highly educated women as are now offered by the leading professions, the schools will lose from their ranks those whose social and educational attainments would, after training, fit them for leadership in the nursing world.

At present nurses are drawn from all classes, with a widely differing standard of education, and as long as the " sense of vocation " has impelled the choice and maintained the enthusiasm a " true nurse " has been evolved. And the sense of vocation must never be divorced from the art of nursing. It is the touchstone of true success in the profession. The retrograde movement of later years may be attributed to the subordination of this " sense " to the desire for a professional career—a mere means of livelihood or occupation. And herein the nursing profession differs from others, and leads to much misunderstanding in the lay mind, which discounts the necessary self-sacrifice and self-denial of such a vocation.

The advance of science has widened the range of a nurse's outlook and elaborated a more perfect technique which, for successful co-operation, demands from the nurse a more intelligent appreciation of detail with a mental alertness that can only be built up on a sound educational basis, begun in early years and continued throughout her training. The previous education is not necessarily one of amassed knowledge of the ordinary school subjects, but one that has rendered the mind receptive of knowledge, delicate in perception, and fastidious in production. This type of mind is not formed by the county and Board schools nor by the home life of many who seek entrance into the ranks of the nursing world and who do, for the most part, fill the training-schools of the workhouse infirmaries and allied institutions. Nor can these institutions do otherwise till candidates of a wider social life and better standard of education be found to recruit their ranks and benefit by the magnificent work and unexampled experience to be gained in them. (How perfectly would the religious orders minister to the needs of our permanently infirm and sick!)

It may be argued that a candidate educated in L.C.C., or similar, schools would find little difficulty in maintaining the educational standard that might in future be set up by the large training-schools, and would compete favourably in examinations with a candidate who has had private tuition only—*i.e.* home education without competition, and perhaps of a somewhat perfunctory type; but the stereotyped mind of the one will present difficulties in training that the more plastic mind of the other does not, and the additional refinement of the latter is a distinct asset in acquiring the fine art of nursing.

The rank and file are needed to suit the diversified branches of the profession—all grades meeting on the common ground of devotion to their calling—but many are manifestly unequipped by education or nature to be organisers and teachers, or to fill the higher branches of administrative work. Nevertheless, these should find in their training-school a wise system of theoretical instruction aiming at developing the mental powers as well as perfecting the practical technique.

The women of High School and University education, who desire to devote themselves to nursing the sick, should be able to find a training suitable to their greater powers, and it would seem that the premier training-schools might advisably make this their aim by providing a curriculum that should offer in addition to the ward experience and the courses of lectures at present in vogue a more systematised and comprehensive instruction spread over the three years of work and adapted to the increasing knowledge and capacity of the nurse.

A preliminary training-school, under wise guidance, is an invaluable asset in preparing the soil for readily assimilating ward experience and in inculcating method, self-discipline, and the professional " sense " which a nurse often gains so hardly in the hospital ward.

The first year, being the most critical in a nurse's life, when she tests her aspirations, forms her habits and lays the groundwork of the later superstructure, should preferably be devoted to a careful course of instruction, covering the ground of practical nursing, its theory and methods, with the necessary but limited amount of anatomy and physiology—subjects taught in the second year by members of the surgical and medical staff—that will actually bear on the practical application of the point at issue. The system followed should aim at developing individual thought and effort, while preserving the corporate sense and uniformity in practice; rather stimulating the interest and " desire to know " than requiring the memorising of facts that may be so many " dry bones."

In this year the nurse may be trained to collate her reasoned facts, judge of essential points, acquire methods of arrangement and ordered thought, test her " nursing sense " and powers of observation, and so benefit her ward as her thought manifests in act.

Much laborious work in the future may be spared to her when the more serious studies furnished by lectures from members of the medical and surgical staff are undertaken in the second and third years of her training.

In the early development of such a scheme two tendencies must be met: the self-assurance of partial knowledge shown by the nurse of superficial mind, and the reluctance to assume responsibility by the more serious and humble-minded nurse who has glimpsed possibilities and dreads disaster. These discouragements will probably disappear when all in authority and in training have passed through the same portal.

The climax of her efforts must inevitably be the final test of her general efficiency by a comprehensive and searching examination both in theory and practice of nursing, giving full weight to evidence accumulated throughout the three years of her loyalty, her response to discipline, her powers of organisation, her training of juniors, her influence among her colleagues, in fact the character she has built up to enhance or detract from the knowledge and technique she has gained in her three years of training and her real contribution to the prestige of the nursing profession.

The process of reconstruction throughout the many branches of the nursing world must necessarily be slow, till the whole body is permeated with the influence and receives the practical support of those who have passed the test and reached the desired standard.

X.

